# Supplementary material for: Design, Synthesis, and Validation of a Novel [11C]Promethazine PET Probe for Imaging Abeta Using Autoradiography
Source: Molecules. 2021 Apr 10;26(8):2182. doi: 10.3390/molecules26082182 (PMC8070574; doi:10.3390/molecules26082182)
Supplement: Supplementary file 1 [file molecules-26-02182-s001.pdf]

Supplementary data

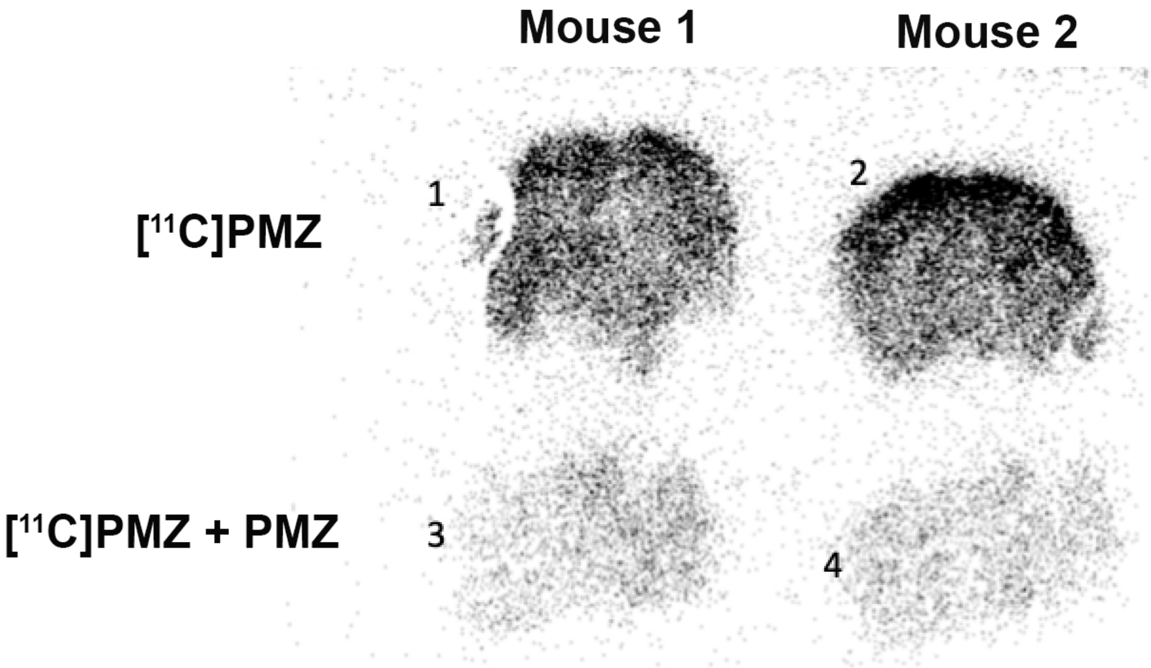

Figure S1. Assessment of the specificity of the [^{11}C]PMZ probe using hippocampal brain tissues of 5XFAD mice.
